# *cchsflow*: an open science approach to transform and combine population health surveys

**DOI:** 10.17269/s41997-020-00470-8

**Published:** 2021-03-24

**Authors:** Warsame Yusuf, Rostyslav Vyuha, Carol Bennett, Yulric Sequeira, Courtney Maskerine, Douglas G. Manuel

**Affiliations:** 1 Ottawa Hospital Research Institute, Civic Campus, ASB 2-012, 1053 Carling Avenue, Ottawa, ON K1Y 4E9 Canada; 2 Department of Family Medicine, University of Ottawa, Ottawa, Ontario Canada; 3 ICES, Ottawa and Toronto, Ottawa, Ontario Canada; 4 Statistics Canada, Ottawa, Ontario Canada; 5 School of Epidemiology, Public Health and Preventive Medicine, University of Ottawa, Ottawa, Ontario Canada

**Keywords:** Health surveys, Data analysis, Data science, Population health, Enquêtes de santé, analyse des données, science des données, santé de la population

## Abstract

**Setting:**

The Canadian Community Health Survey (CCHS) is one of the world’s largest ongoing cross-sectional population health surveys, with over 130,000 respondents every two years or over 1.1 million respondents since its inception in 2001. While the survey remains relatively consistent over the years, there are differences between cycles that pose a challenge to analyze the survey over time.

**Intervention:**

A program package called *cchsflow* was developed to transform and harmonize CCHS variables to consistent formats across multiple survey cycles. An open science approach was used to maintain transparency, reproducibility and collaboration.

**Outcomes:**

The *cchsflow* R package uses CCHS survey data between 2001 and 2014. Worksheets were created that identify variables, their names in previous cycles, their category structure, and their final variable names. These worksheets were then used to recode variables in each CCHS cycle into consistently named and labelled variables. Following, survey cycles can be combined. The package was then added as a GitHub repository to encourage collaboration with other researchers.

**Implication:**

The *cchsflow* package has been added to the Comprehensive R Archive Network (CRAN) and contains support for over 160 CCHS variables, generating a combined data set of over 1 million respondents. By implementing open science practices, *cchsflow* aims to minimize the amount of time needed to clean and prepare data for the many CCHS users across Canada.

## Résumé

**Contexte:**

L’Enquête sur la santé dans les collectivités canadiennes (ESCC) est l’une des plus grandes enquêtes transversales sur la santé de la population, avec plus de 130 000 sondés tous les deux ans et plus de 1,1 million de sondés depuis son début en 2001. Tant que l’enquête reste relativement cohérent, il y a des différences entre des cycles qui posent une challenge majeure pour analyser l’enquête au fil du temps.

**Intervention:**

Un paquet de programme appelé *cchsflow* a été développé pour transformer et harmoniser les variables CCHS aux formats cohérents à travers plusieurs cycles de sondage. Une approche de science ouverte était utilisée pour maintenir la transparence, la reproductibilité et la collaboration.

**Résultats:**

Le paquet *cchsflow* R développé utilisait les données d’enquête de l’ESCC entre 2001 et 2014. Les feuilles de calcul ont été créées pour identifier des variables, leurs noms dans des cycles précédents, leurs structures de catégories et leurs noms de variables finales. Ces feuilles de calcul ont ensuite été utilisées pour recoder les variables dans chaque cycle de l’ESCC pour générer les ensembles de données harmonisés qui peuvent être combiner dans un ensemble de données constamment étiqueté pour l’analyse. Le paquet a ensuite été ajouté comme un entrepôt de GitHub pour encourager la collaboration avec les autres chercheurs.

**Implication:**

Le paquet *cchsflow* a été ajouté au Comprehensive R Archive Network (CRAN) et contient un appui pour plus de 160 variables de l’ESCC, générant un ensemble de données de plus d’un million de sondés. En exécutant les pratiques de sciences ouvertes, *cchsflow* vise à minimiser le temps requis pour nettoyer et préparer les données pour les plusieurs utilisateurs du CCHS à travers le Canada.

## Introduction

You are a public health epidemiologist who would like to report the change in body mass index (BMI) in your health unit over the past 15 years. You review the codebook for the Canadian Community Health Survey (CCHS) and note that BMI is collected. BMI *seems* like a straightforward measure that is routinely collected worldwide (Statistics Canada 2001). Indeed, BMI is included in all CCHS cycles. You examine the documentation and find the variable HWTAGBMI in the CCHS 2001 corresponds to body mass index, but that in other cycles, the variable name changes to HWTCGBMI, HWTDGBMI, HWTEGBMI, etc. On reading the documentation, you notice that some cycles round the value to one decimal, whereas other cycles round to two digits. Furthermore, some cycles don’t calculate BMI for respondents under the age of 20 or over the age of 64 years. Also, some cycles calculate BMI only if height and weight are within specific ranges.

After spending hours on the task, you talk with a colleague in a neighbouring health unit. They did the same task a few years ago. You share your Stata code by email and compare notes, only to realize that you both had different approaches, each with errors.

A process called *cchsflow* was created to minimize the amount of time public health epidemiologists and others spend cleaning and transforming CCHS variables across multiple survey cycles. An open science approach was sought for the development of *cchsflow*. Open science is the movement to improve research reproducibility, accessibility, and collaboration (Ross and Krumholz 2013). Public health practice strives for these qualities and, therefore, the field can potentially benefit from the same tools that are used to support open science. An example of the open science toolkit is versioning software and cloud-based repositories such as GitHub and GitLab that allow people to collaborate and share programming code.

Currently, *cchsflow* harmonizes 160 variables for 1,092,951 survey respondents of the CCHS Public Use Microdata File (PUMF) from 2001 to 2014. *cchsflow* uses open science tools to allow users the ability to contribute to the package, including making suggestions and requests, and to identify errors. People can also “fork” the package, meaning they can use the *cchsflow* approach to harmonize other databases. *cchsflow* uses R language package since R is the most commonly used open statistical programming language. The core of *cchsflow*, however, are reference files that could be used in other programming languages.

Even this paper was created using the open science principles. This paper was written using R Markdown—a notebook that allows R code to be executed within a document. Both the *cchsflow* R package and this paper’s notebook are available on GitHub which allows readers to make comments and suggestions or note errors. Readers can execute or modify all examples in this paper in R.

## Background

### Cleaning and transforming CCHS data

Data cleaning, including transforming variables into harmonized or common variables, is typically the most time-consuming part of data analyses. According to Dasu and Johnson, 80% of data analysis is spent on data cleaning (Dasu and Johnson 2003). With the CCHS, data cleaning and harmonization issues arise when combining CCHS surveys. Currently, there is no standardized method or tool used to combine CCHS survey cycles. Health units across Canada that use the CCHS do their own data cleaning and preparation, taking time away from other data analysis.

### Open science and its benefits to public health practice

Open science is defined as “transparent and accessible knowledge that is shared and developed through collaborative networks” (Vicente-Saez and Martinez-Fuentes 2018). Included in open science is: open data, data that are publicly accessible such as the CCHS with Statistics Canada’s new Open Licence (Statistics Canada 2020); open source, the use of open access programs such as data science languages including R, Python and Julia; and open methodology, program code that is publicly accessible and shared through online repositories (McKiernan et al. 2016; Stodden et al. 2013). In public health, there has been a marked trend toward open data and sharing code—notably during the COVID-19 pandemic (Moorthy et al. 2020).

Adopting open science practices comes with well-described benefits (Donoho 2017; Hicks and Irizarry 2018). McKiernan et al. found that open science is associated with increased research exposure in both media and in citations; and an increase in collaboration, funding, and job opportunities (McKiernan et al. 2016). For public health professionals, an open science approach and toolkit facilitates collaboration as it allows for data and coding methods to be shared between different health units. Additional benefits include improved transparency, accessibility, and efficiency, reduced coding errors, and faster analyses. As in other sectors, public health practitioners can use open science tools to potentially improve and compress many time-consuming, repetitive, and inconsistent analysis tasks. In light of the COVID-19 pandemic, open science allows public health researchers to quickly aggregate and analyze data across many health units to guide policy-makers in making informed public health decisions.

## Methods

*cchsflow* follows the approach of the Open Source Initiative and open software for research. The developers of *cchsflow* are public health researchers who collaborate with federal, provincial and local public health units. *cchsflow *was developed following publication of several peer-reviewed reports created with Public Health Ontario, ICES and the Ontario Public Health Association (Journal of Open Source Software 2018; Manuel et al. 2012; Open Source Initiative 2020). One of the developers of *cchsflow* (DGM) is a part-time employee at Statistics Canada. The *cchsflow* package is not a Statistics Canada product, nor is the package supported by Statistics Canada. However, analysts at Statistics Canada use *cchsflow* and have contributed to variable transformations.

The package currently supports the first 10 cycles of the CCHS PUMF surveys from 2001 to 2014, in which the variables of each were harmonized and transformed to use the same set of variables. In *cchsflow*, variables were renamed to the variable names used in CCHS cycles from 2007 to 2014.

Many variables in *cchsflow* are used in peer-reviewed studies of our development team and other researchers (Manuel et al., 2012, 2016, 2018, 2020a). Occupation variables, for example, were incorporated from peer-reviewed occupation studies (Nowrouzi-Kia et al. 2019). Depression variables are an example of variables for which there was not consistent use in peer-reviewed literature, but which were added in consultation with mental health researchers. Open discussion with the mental health researchers is included in the package development (https://github.com/Big-Life-Lab/cchsflow/pull/64 ). Anyone can participate in the discussions when new variables are added. *cchsflow* was created in R with provisions to support other program languages such as Stata or SAS.

### Selection of variables

Variables included in *cchsflow* fall into three categories: health behaviours, socio-demographic information, and health status. At the time of writing, there are 160 variables, 30 subjects and 6 sections. There are provisions and instructions on how users can contribute or request the addition of new variables.

Health behaviours variables include smoking, alcohol, diet, and physical activity (Conner and Norman 2017). There are derived variables such as smoking pack-years (pack_years_der) that are not available in the original CCHS data files.

Socio-demographic variables include age, sex, immigration status, country of birth, time spent in Canada, ethnicity, education (individual and highest family), income (adjusted for province and inflation), home ownership, and marital status. Harmonized occupation variables were created (LBFA_31A, LBFA_31A_A and LBFA_31A_B) by reviewing studies that used the CCHS to study occupation (Nowrouzi-Kia et al. 2019). References to these papers are included in the notes section of the variable transformation.

Health status variables include chronic disease, the Health Utility Index, need for help for activities of daily living (ADL), mental health, and other measures. There is a new derived variable for the number of ADL requiring assistance (ADL_score_5) that is not available in the original CCHS data.

### Variable mapping

CCHS variables were transformed across 10 survey cycles. For many variables, the only difference between cycles was their variable name. As such, only a name change was required to standardize a variable across the 10 cycles.

Changes in the number and type of categories were also common. For example, in the 2001 and 2003 CCHS survey cycles, there were 15 age categories; while in CCHS survey cycles from 2005 to 2014, there were 16 age categories. There were two options for such variable category changes. The first option was to create a harmonized variable by collapsing categories into common forms. The second option was to maintain separate variables. For age, a third option was also added to maximize age information by deriving a new continuous age variable, one that takes the midpoint of each age category for all cycles.

There were also changes to question wording, missing categories, and inclusion and exclusion criteria. Variables were not included in all cycles or all health regions. Harmonized variables were included when there was a consensus among developers that the differences across cycles were small. *Notes* were included when any difference was identified, with a default to print all notes during transformations.

### Transformation of variables through specification worksheets

Two worksheets are included in the *cchsflow* packages that contain variable information and metadata: *variables.csv* specifies all the variables in the package and *variable_details.csv* specifies CCHS data that contain the variables, the variable type, and the category structure.

*cchsflow* was created using the *recodeflow* R package—also developed by the authors. Within *recodeflow*, the *rec_with_table()* function—short for “recode with table”—transforms variables. *rec_with_table()* uses the two worksheets to create a transformed data from a CCHS cycle. Once all CCHS survey cycles have been transformed, they can be combined to create one large transformed data set that spans across the 10 CCHS survey cycles. The two CSV worksheets also have variable labels and other metadata that can be added to the data using the *rec_with_table()* function.

### Derived variables

CCHS includes derived variables that were created using multiple responses and variables. BMI is an example of an original CCHS derived variable that was calculated using self-reported height and weight. Several new derived variables were included, such as smoking pack-years, binge drinking, and diet pattern (Manuel et al. 2016). There are provisions and instructions for adding additional derived variables.

### Documentation

Open source, web-based documentation is available at https://big-life-lab.github.io/cchsflow/, and includes a searchable reference of all transformations, vignettes with examples of how to perform transformations, collaboration principles, and a development roadmap.

## Results

**Fig. 1:**
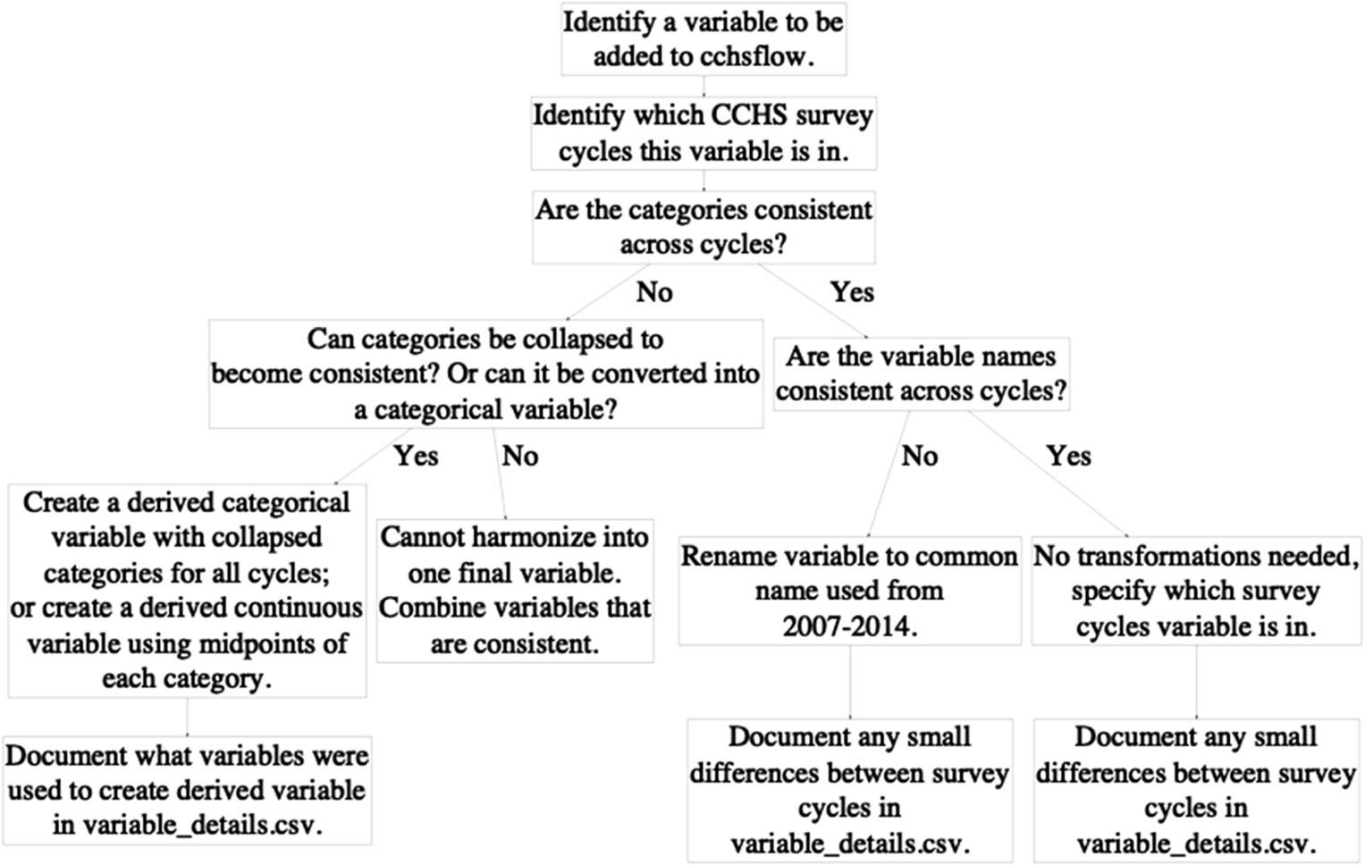
Flowchart of how CCHS variables were added to *cchsflow*. Users can add variables using the same approach

**Fig. 2:**
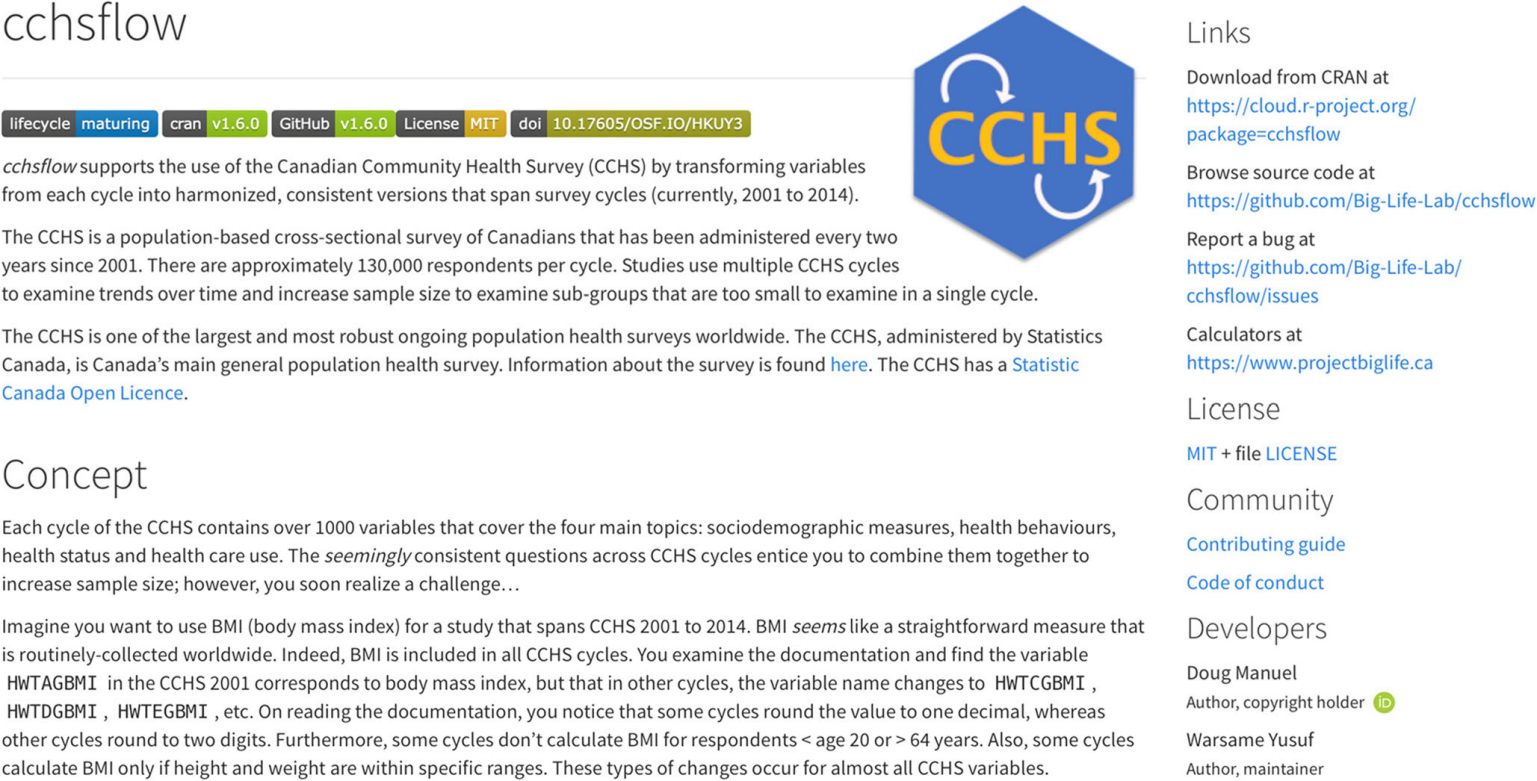
The homepage for the *cchsflow* website

The *cchsflow* package is available on the Comprehensive R Archive Network (CRAN), a network of servers that contain documentation for R packages (Manuel et al. 2020c). The package contains the following items: the *variables.csv* worksheet, the *variable_details.csv* worksheet, the various functions, and subsets of 200 respondents for each CCHS cycle. Figure 1 illustrates how variables were added to *cchsflow*, while Figure 2 illustrates the homepage of the *cchsflow* package at https://big-life-lab.github.io/cchsflow/. 

**Fig. 3:**
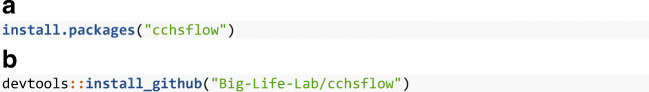
**a** The command line to install the *cchsflow* package that is currently saved on CRAN. **b** The command line to install the development version of *cchsflow* from GitHub

Figure 3a illustrates the command line to install the CRAN version of *cchsflow*, while Figure 3b illustrates the command to install the development version of *cchsflow*, which is a more up-to-date version of the package.

### Recode with table

**Fig. 4:**
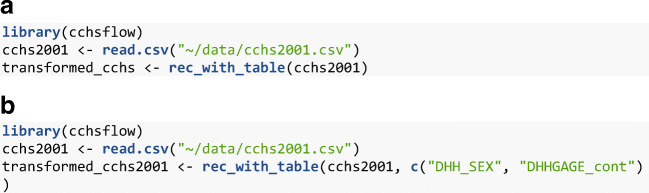
**a** The command lines to load the *cchsflow* package, load the 2001 CCHS PUMF data and then transform all the variables in the worksheets using the *rec_with_table()* function. **b** The command lines to transform the sex & age variables using the *rec_with_table()* function

The *rec_with_table()* function is used to recode or transform variables based on the information from the two specification worksheets. The function has the ability to transform an entire data set, or a subset of variables. Figure 4a illustrates how to load the *cchsflow* package and the 2001 CCHS data, and then transform all variables in *cchsflow* to their harmonized version. The *cchsflow* package comes with a subsample of CCHS data for 2001 to 2014 versions, made possible with Statistics Canada new Open Licence (Statistics Canada 2020). Figure 4b illustrates how to transform a subset of variables from the 2001 survey cycle.

## Discussion

*cchsflow *harmonizes and transforms CCHS data from 2001 to 2014 (Manuel et al. 2020c). *cchsflow* provides public health epidemiologists and others the ability to more robustly analyze over 1 million respondents across a 13-year period to examine trends in health indicators. The use of an open science approach improves collaboration, transparency, and efficiency when transforming variables. The package allows public health professionals who use CCHS to spend less time on data cleaning and spend more time on analysis such as surveillance and health status reporting.

### Comparison to other projects

A consistent approach to calculate health indicators is a long-standing public health goal. *cchsflow* uses an open science approach to build from and support several health-related indicator and harmonization projects that use CCHS data, including the Canadian Institute for Health Information indicator library, the Public Health Agency of Canada health inequality reports, and Ontario’s Public Health Indicator Working Group (Association of Public Health Epidemiologists in Ontario 2018; Canadian Institute for Health Information 2020; Pan-Canadian Public Health Network 2018). These initiatives typically include the definition of indicators, but it is uncommon to publish how to calculate indicators using CCHS data, especially across CCHS cycles.

Observational Health Data Sciences and Informatics (OHDSI) is an open science network that creates a common dictionary and software tools to support studies across different information systems (ODESI 2020). The focus of OHDSI is hospital data. Investigators in different hospitals generate their own code to harmonize their hospital data into common, standard definitions.

*cchsflow* facilitates the use of CCHS metadata that come with the survey but are not commonly used by public health practitioners (Manuel and Fisher 2019). The CCHS comes with Data Documentation Initiative (DDI) metadata. DDI metadata are used worldwide for over ten thousand different surveys and research projects (Data Documentation Initiative (DDI) 2020). There are also initiatives such as Maelstrom that are used by other Canadian health surveys to improve the use of metadata (Bergeron et al. 2018). Metadata are increasingly recognized as helpful data infrastructure to support open science and data harmonization. Metadata are “data about data” and include information about variable and category labels, variable types, and provenance (how the data were collected and transformed) (McGilvray 2008).

Barriers to using metadata in public health include the lack of well-organized metadata in public health data and the lack of metadata analysis tools such as *cchsflow*. It is commendable that DDI documents are included with CCHS, but not all metadata are included or consistent. Variable transformation is robustly supported in newer versions of DDI that are not yet available for the CCHS. *cchsflow* uses DDI documents to create the worksheets with the added benefit of harmonizing and transforming metadata across CCHS cycles. *cchsflow* also supports the use of Predictive Model Markup Language (PMML) (Grossman et al. 1999). The Project Big Life team uses *cchsflow*’s PMML metadata in public health planning tools (Manuel et al. 2020a).

### Limitations and challenges

While the CCHS has many consistent variables across survey cycles, there are differences between cycles that can be irreconcilable or difficult to harmonize. Within *cchsflow*, variables with irreconcilable differences were either transformed into a new derived variable or kept as separate variables that can only be used in select cycles. Along with variables with differences, there are variables in *cchsflow* that were not asked in all CCHS cycles. This means that for some variables, data do not span across the length of the CCHS cycles available in *cchsflow*. A possible solution is to impute missing variables, where missing data are replaced with values based on other respondents and responses to other variables.

Care must be taken to understand how specific variable transformation and harmonization with *cchsflow* affects each use of CCHS data. Across survey cycles, almost all CCHS variables have had at least some change in wording and category responses. Furthermore, there have been changes in survey sampling, response rates, weighting methods and other survey design changes that affect responses. Combining CCHS data across survey cycles will result in misclassification error and other forms of bias that affect studies in different ways.

### Collaboration with other users

Collaboration is facilitated using GitHub, the most popular online code repository with over 45 million users. GitHub is based on the Git version-control system which, in turn, is a cornerstone of open software development (Dabbish et al. 2012).

The open-access approach *cchsflow* allows users to add other CCHS variables that might benefit others. There is full transparency on how the package was developed with the entire source code for the package publicly accessible. Along with being transparent, sourcing the *cchsflow* package on GitHub offers users of the package the opportunity to provide feedback on how to further improve the package. In the issues section of the GitHub repository, users can submit bug reports where they can identify issues they are encountering while using the package. Users can also request variables to be added or add new variable transformations themselves. New variables are added using a “pull request” that is then reviewed by the package maintainers before being merged with the main *cchsflow* package. All *cchsflow* documentation (including this paper write-up) are also open access and available on the GitHub repository.

GitHub provides benefit to users in that it allows them an opportunity to implement better practices in their own code (Dabbish et al. 2012). The implementation of GitHub in the development of *cchsflow* allows public health professionals across Canada to collaborate and share potential variables that can be useful for health surveillance and health status reporting. Projects that examine health surveillance and health status reporting such as the Public Health Agency of Canada health inequality reports (Pan-Canadian Public Health Network 2018) can benefit from *cchsflow*’s repository of harmonized variables.

### Roadmap

A roadmap, also known as next steps or future plans, is recommended for open software projects. *cchsflow* includes a roadmap and milestones on the project website. At the time of writing, the roadmap includes adding the “share” version of CCHS that is used in Statistics Canada Regional Data Centres and other settings, the ability to compare variable frequency across survey cycles, and improved metadata support. *cchsflow* has been forked by related projects to support other data sets. The expanded use of *cchsflow* for related projects is a hallmark of open science and a demonstration of how open science leads to expanded science and public health resources.

## Conclusion

*cchsflow*’s open science approach allows public health professionals to collaborate and share their work with other colleagues, saving time spent on recoding and cleaning health data. By implementing open science practices, *cchsflow* aims to minimize the amount of time needed to clean and prepare CCHS data for the many CCHS users in health units across Canada.
